# Swimming by reciprocal motion at low Reynolds number

**DOI:** 10.1038/ncomms6119

**Published:** 2014-11-04

**Authors:** Tian Qiu, Tung-Chun Lee, Andrew G. Mark, Konstantin I. Morozov, Raphael Münster, Otto Mierka, Stefan Turek, Alexander M. Leshansky, Peer Fischer

**Affiliations:** 1 Max Planck Institute for Intelligent Systems, Heisenbergstrasse 3, Stuttgart 70569, Germany; 2 Institute of Bioengineering, Ecole Polytechnique Fédérale de Lausanne (EPFL), Lausanne CH-1015, Switzerland; 3 Faculty of Chemical Engineering, Technion—Israel Institute of Technology, Haifa 32000, Israel; 4 Institute of Applied Mathematics (LS III), TU Dortmund, Vogelpothsweg 87, Dortmund 44227, Germany; 5 Technion Autonomous Systems Program (TASP), Haifa 32000, Israel; 6 Institut für Physikalische Chemie, Universität Stuttgart, Pfaffenwaldring 55, Stuttgart 70569, Germany

## Abstract

Biological microorganisms swim with flagella and cilia that execute nonreciprocal motions for low Reynolds number (Re) propulsion in viscous fluids. This symmetry requirement is a consequence of Purcell’s scallop theorem, which complicates the actuation scheme needed by microswimmers. However, most biomedically important fluids are non-Newtonian where the scallop theorem no longer holds. It should therefore be possible to realize a microswimmer that moves with reciprocal periodic body-shape changes in non-Newtonian fluids. Here we report a symmetric ‘micro-scallop’, a single-hinge microswimmer that can propel in shear thickening and shear thinning (non-Newtonian) fluids by reciprocal motion at low Re. Excellent agreement between our measurements and both numerical and analytical theoretical predictions indicates that the net propulsion is caused by modulation of the fluid viscosity upon varying the shear rate. This reciprocal swimming mechanism opens new possibilities in designing biomedical microdevices that can propel by a simple actuation scheme in non-Newtonian biological fluids.

## Abstract (web summary)

Operating devices that can navigate biomedical fluids and tissues is one of the ultimate goals of microrobotics. Whilst the current designs are restricted to non-reciprocal actuations, Qiu *et al*. report swimming via simple reciprocal motion by exploiting the non-Newtonian rheology of viscous fluids.

## Graphical abstract

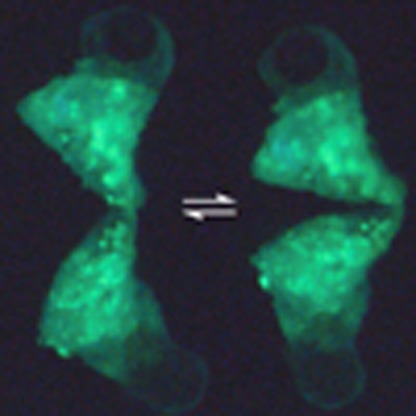

Motility is important for the survival of many organisms. At the length scale of primitive life forms, such as bacteria and other microorganisms, locomotion presents a different set of challenges compared with those encountered by macroscopic organisms. Most microorganisms live in fluid environments where they experience a viscous force that is many orders of magnitude stronger than inertial forces. This is known as the low Reynolds number (Re) regime (Re*≪*1) characterized by instantaneous and time-reversible flows that are described by the time-independent Stokes equation. A consequence of this is the ‘scallop theorem’, stated by Purcell in his 1976 paper on ‘Life at low Reynolds number’^1^. If a low-Reynolds number swimmer executes geometrically reciprocal motion, that is a sequence of shape changes that are identical when reversed, then the net displacement of the swimmer must be zero, if the fluid is incompressible and Newtonian.^2^ In Purcell’s own words, ‘Fast, or slow, it exactly retraces its trajectory, and it’s back where it started’^1^.

Locomotion at low Re therefore generally requires nonreciprocal actuation of the swimmer. In nature, microorganisms break time-reversal symmetry with rotating helices^3^ and cilia that show flexible oar-like beats^4^. Inspired by nature, similar swimming strategies have been utilized to propel artificial microswimmers. These include helically shaped micropropellers that use rigid chiral structures to break symmetry under nonreciprocal unidirectional rotation^5^,^6^,^7^,^8^,^9^,^10^. As a helix rotates about its long axis, the coupling between rotational and translational motion leads to propulsion at low Re. However, reciprocal actuation of a helix does not generate directed propulsion but only enhanced diffusivity^11^,^12^. A few flexible microswimmers have also been experimentally demonstrated, including a microswimmer that is based on a chain of superparamagnetic beads and actuated by a magnet^13^, a biohybrid elastic microswimmer made of elastic filament and actuated by cardiomyocytes^14^, as well as model swimmers that use flexible tails^15^,^16^,^17^,^18^. However, in order to break reciprocity, these swimmers require relatively complex fabrication processes and/or actuation mechanisms.

Propulsion of artificial microswimmers has mainly been demonstrated in Newtonian fluids, while low Re propulsion in non-Newtonian fluids remains relatively unexplored^19^, even though most biological fluids are non-Newtonian. In fact, most of the fluids in the human body are non-Newtonian viscoelastic media^20^, for example, sputum, mucus and vitreous humour, with many of them, for example saliva, blood and synovial fluid, showing shear thinning behaviour^21^. Since the scallop theorem no longer holds in complex non-Newtonian fluids, it follows that it should be possible to design and build novel microswimmers that specifically operate in these complex fluids.

Fluid elasticity can either enhance^18^,^22^,^23^ or retard^24^ propulsion in non-Newtonian fluids. Recent theoretical work on a beating flagellum in a nonlinear viscoelastic Oldroyd-B fluid^25^ as well as on a reciprocal sliding sphere swimmer in a shear thinning fluid^26^ also suggests that propulsion is achievable by reciprocal motion, in which backward and forward strokes occur at different rates. Similarly, the elasticity of the fluid has enabled low Re propulsion of oscillating asymmetric dimers^27^. However, low Re locomotion of a true reciprocal motion microswimmer propelled by periodic body-shape changes has not been reported previously.

Here we build and actuate single-hinge micro- and macroswimmers that move in the manner of the ‘scallop’ described by Purcell^1^. The dimensions of the micro-scallop are submillimetre, approaching the size regime of relevance for noninvasive exploration of, for instance, blood vessels. Even though they are restricted to reciprocal motion, the scallops achieve low Re propulsion by using a time-asymmetric stroke pattern, and exploiting the strain rate-dependent viscosity of shear thickening and shear thinning fluids. Precise control over the macro-scallop permits quantitative comparison with our numerical modelling results and analytical theory for the propulsion mechanism. Excellent agreement between experiment and theory is found, confirming that the net propulsion is caused by the differential apparent fluid viscosity under asymmetric shearing conditions. The results demonstrate that, despite the scallop theorem, in biologically relevant fluids simple actuation schemes can generate propulsion.

## Results

### Design and actuation of the micro-scallop

**Figure 1:**
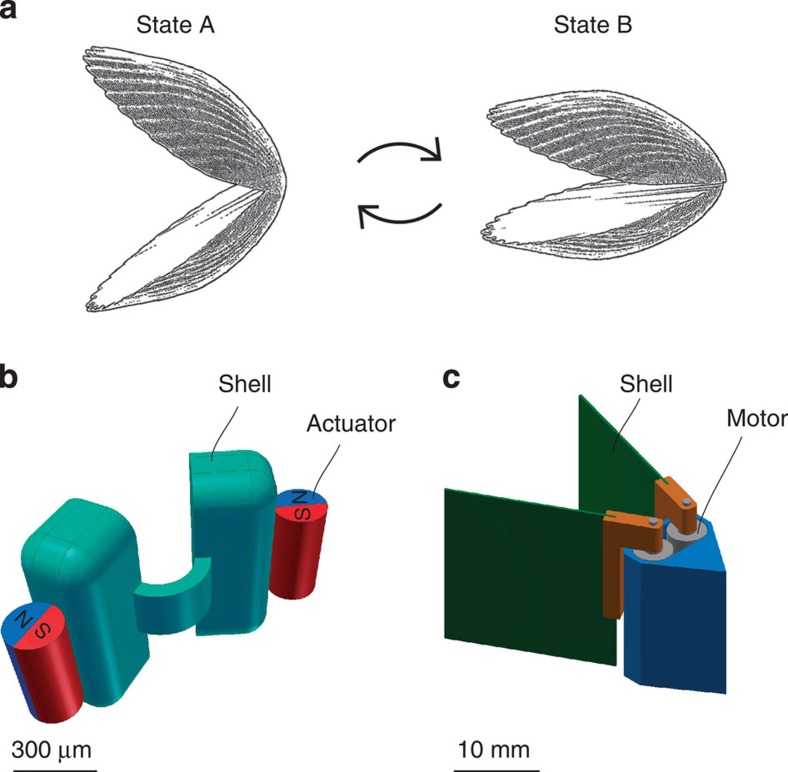
Schematic drawing of the scallop swimmers. (**a**) Schematic drawing of Purcell’s scallop with reciprocal motion^1^. (**b**) 3D model of the submillimetre size ‘micro-scallop’. (**c**) 3D model of the centimetre size ‘macro-scallop’ for quantitative comparison with theory.

**Figure 2:**
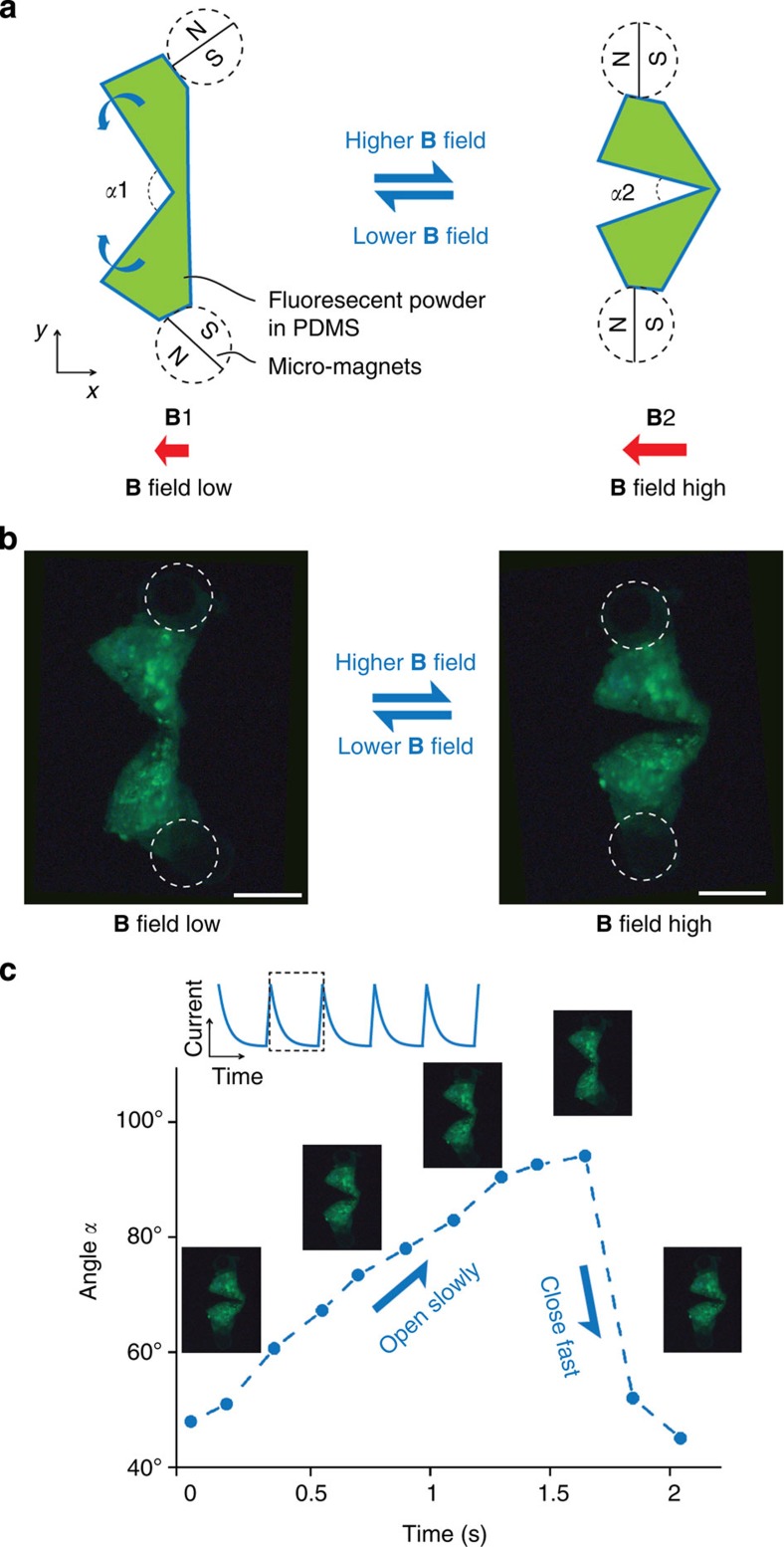
Actuation mechanism of the micro-scallop. (**a**) Schematic drawing of the micro-scallop from top view. The green shapes illustrate the opening and closing shape change of the micro-scallop when actuated by an external magnetic field. The shape is a function of the magnetic force aligning the magnetic axes of the two permanent micromagnets and a restoring force because of the induced stress in the PDMS structure. The angle between the two shells *α* can therefore be controlled by the magnitude of the external field. (**b**) Top view (microscope image) of the micro-scallop under ultraviolet illumination. The positions of the micromagnets are illustrated by white dashed circles. Scale bar, 200 μm. (**c**) Time-asymmetric actuation of the micro-scallop. The slow opening and the fast closing cycles are controlled by the external magnetic field, which is generated by an exponentially decaying current (inset). Corresponding images of the micro-scallop during the opening and closing cycles are shown.

Our micro-scallops are constructed from polydimethylsiloxane (PDMS), loaded with phosphorescent pigment, cast into a three-dimensional (3D) printed mold, which permits the use of different materials in a parallel fabrication process. Each micro-scallop consists of two thick (300 μm) shells connected by a thin (60 μm), narrow (200 μm) hinge (Fig. 1b). Rare earth micromagnets (Ø200 μm × 400 μm) are attached to each shell so that when exposed to an external magnetic field the two magnets reorient to align with the field and each other, and thus close the micro-scallop (Fig. 2a, right). When the magnetic field is decreased, the restoring force of the PDMS hinge provides the recovery stroke, opening the micro-scallop (Fig. 2a, left). Alignment with the magnetic field prevents the scallop from pitching and yawing, and ensures that it swims straight. The thick stiff shells and compliant flexible hinge ensure that the deformation is isolated at the hinge. As stated by Purcell: any single-hinge structure can only exhibit reciprocal motion^1^.

The opening angle *α* of the micro-scallop is related to the strength of the applied external field. As shown in Fig. 2c, asymmetric actuation of the two shells is achieved by applying a periodic exponentially decaying current to generate the magnetic field. Typically, a 0.5-Hz waveform was used with a slow ~1.9 s exponential decay, followed by a rapid 0.1 s ramp. Since a gradient-free field is used, the micromagnets do not experience any pulling force, which ensures the net displacement of the microswimmer is because of the propulsion caused by its own shape-changing swimming motions.

### Propulsion of the micro-scallop in a shear thickening fluid

**Figure 3:**
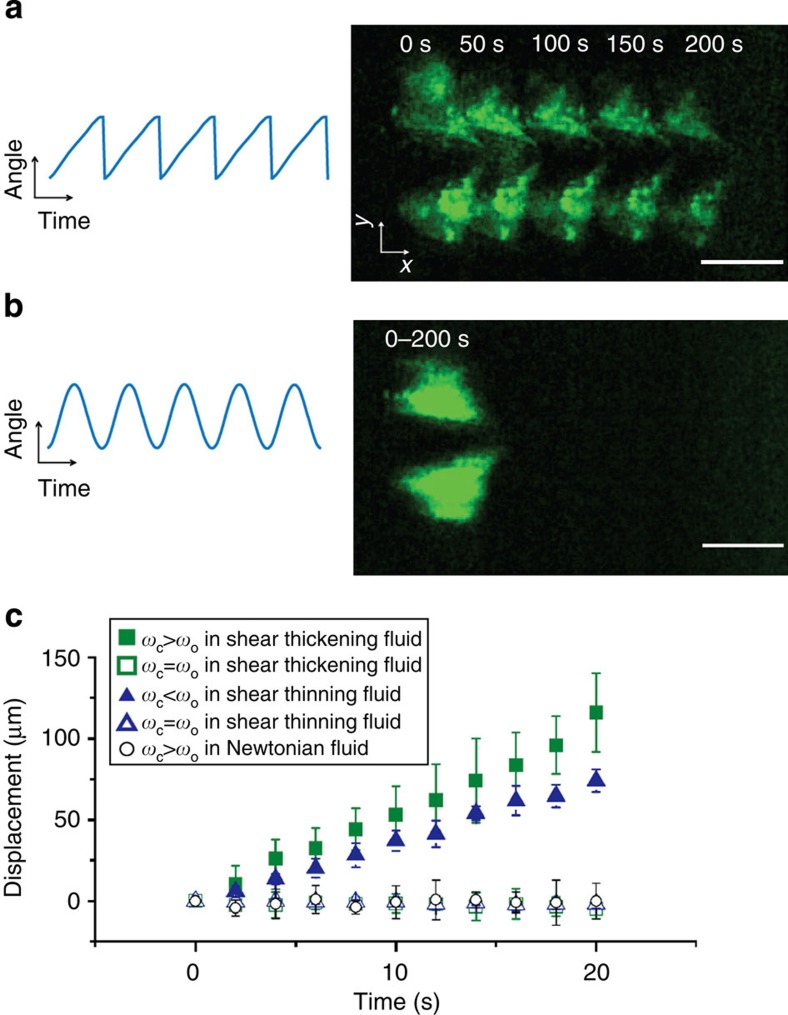
Displacement of the micro-scallop in a shear thickening and a shear thinning fluid. (**a**) Forward net displacement of the micro-scallop in a shear thickening fluid and asymmetric actuation (blue curve). The image is a time-lapse composite picture of five frames at an interval of 50 s, with the net displacement indicated along the *x* direction (see Supplementary Movie 1 upper panel). (**b**) Corresponding image of the micro-scallop in shear thickening fluid with symmetric actuation (blue curve) and no discernable net displacement (see Supplementary Movie 1 lower panel). Scale bar in **a**,**b**, 300 μm. (**c**) Corresponding displacement curves in the shear thickening, shear thinning and Newtonian fluids, respectively. *ω*_c_ and *ω*_o_ are the average angular velocity of closing and opening, respectively. Asymmetric actuations (*ω*_c_>*ω*_o_, solid squares and *ω*_c_<*ω*_o_ solid triangles) result in net displacement in non-Newtonian fluids, while symmetric actuations (*ω*_c_=*ω*_o_, hollow squares and triangles) result in no net displacement in the same fluids (see Supplementary Movie 2 for swimming in shear thinning fluid). Asymmetric actuations (*ω*_c_>*ω*_o_, circles) result in no net displacement in the Newtonian fluid (see Supplementary Movie 3), as stated by the scallop theorem. The error bars correspond to the s.d. of repeated trials.

Forward net displacement of the micro-scallop in a shear thickening fluid (fumed silica in poly(propylene glycol)) was achieved by time-asymmetric actuation of the opening angle as seen in Fig. 3a. Experiments were conducted with the scallop supported at the air–fluid interface (Fig. 3), as well as with micro-scallops that were fully immersed in bulk fluid (Supplementary Fig. 16). Nonreciprocity arising from surface capillary waves in the interface-supported geometry can be ruled out because their phase velocity is too high to be excited by the micro-scallop’s stroke. This is confirmed by the quantitative agreement between the results from the (interface) supported and unsupported (bulk) swimming configurations. Overlaying five frames with the same opening angles taken at 50-s intervals (Fig. 3a) clearly shows net displacement in the *x* direction parallel to the magnetic field direction (Supplementary Movie 1, upper panel). As a control, the micro-scallop in the same fluid was actuated with a symmetric waveform, and, as expected, no net displacement in the *x* direction was observed (Fig. 3b and Supplementary Movie 1, lower panel). Furthermore, asymmetric actuation in a Newtonian fluid (glycerol) also yields no net displacement (circles in Fig. 3c).

**Figure 4:**
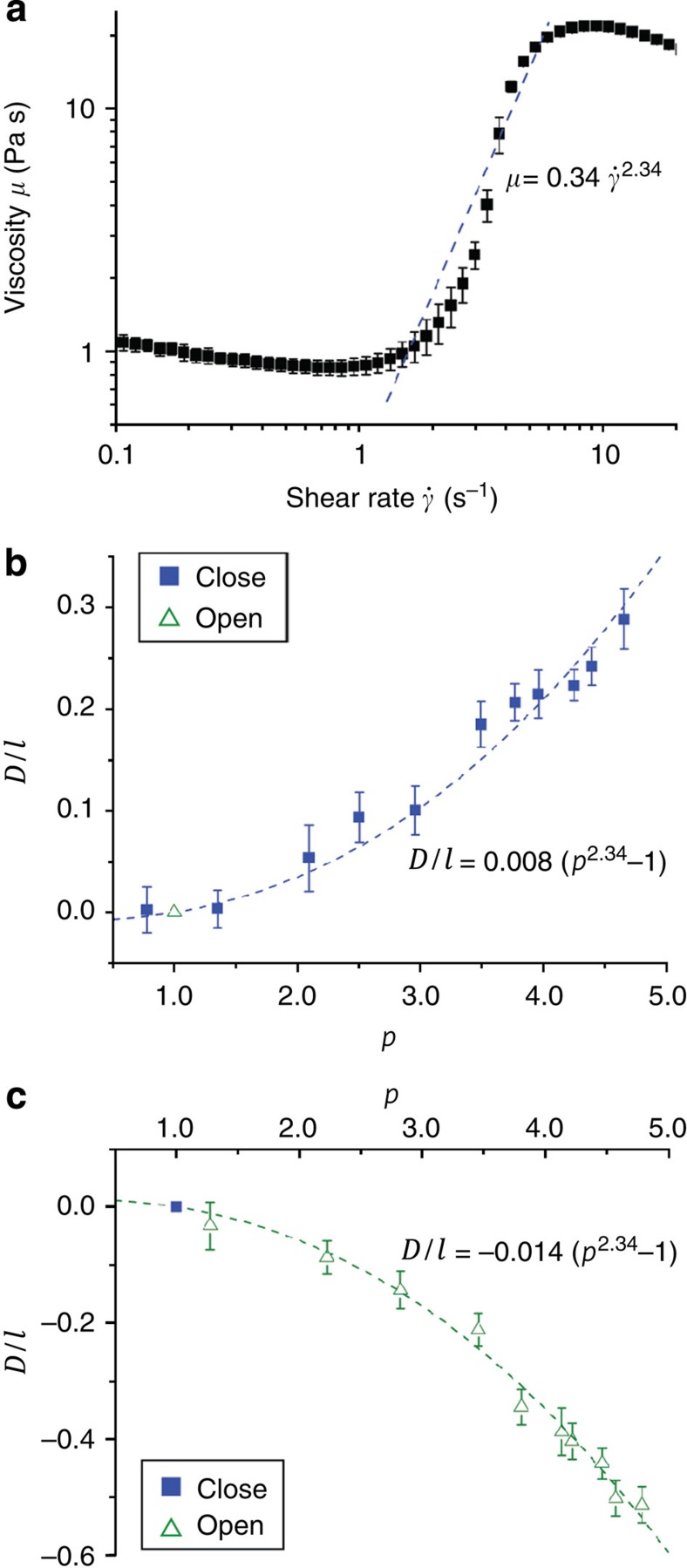
Comparison between theoretical predictions and experimental measurements of scallop swimming. (**a**) Experimentally measured apparent dynamic viscosity of the shear thickening fluid (solid squares). The dashed line shows the power law used to fit the transition to the shear thickening regime. (**b**,**c**) Dimensionless net displacement over one period plotted against the ratio of angular velocities. *D* and *l* are the displacements over one stroke and the characteristic length, respectively. The dashed lines represent the predictions of the scaling theory in Equation (5). When the closing velocities (solid squares) are larger than the opening velocity (hollow triangle), *p*=*ω*_fast_/*ω*_slow_=*ω*_c_/*ω*_o_>1, the swimmer exhibits positive net displacements (**b**). When the opening velocities (hollow triangles) are larger than the closing velocity (solid square), *p*=*ω*_fast_/*ω*_slow_=*ω*_o_/*ω*_c_>1, the swimmer exhibits negative net displacements (**c**). In (**a**–**c**), the error bars correspond to the standard deviations.

Fumed silica particle suspensions were chosen as the shear thickening fluid because of their well-known characteristics^28^ and relatively high viscosity. The dynamic viscosity of the fluid is in the range of 1–22 Pa s (Fig. 4a), and its density is 1051±2 kg m^−3^. The micro-scallop swam for more than 100 μm over 10 periods, and the average velocity was 5.2 μm s^−1^ (3.5% body length per cycle). If we take the characteristic maximum length of the micro-scallop as 1 mm, and the largest forward velocity as 3 mm s^−1^, then the calculated Re=1.4 × 10^−4^–3 × 10^−3^ ≪1. Thus, the microswimmer operates at a very low Re^1^ using only reciprocal motion.

### Propulsion of the micro-scallop in a shear thinning fluid

Propulsion of the micro-scallop operated with a reciprocal but asymmetric actuation sequence is also achieved in hyaluronic acid, a shear thinning solution found in a number of biological systems. In the shear thinning fluid, the micro-scallop only moves forward when the opening–closing cycle is opposite to that of the shear thickening fluid. Now, fast-opening followed by a slow-closing step gives forward propulsion. About 65 μm displacement was covered by the micro-scallop in 10 periods, corresponding to an average velocity of 3.8 μm s^−1^ (2.5% body length per cycle). As in the shear thickening medium, the micro-scallop showed no significant forward displacement when the opening and closing cycles were symmetric, as is expected (see Supplementary Movie 2).

### Analytical theory of propulsion

We consider a simple model of a flapping, Purcell scallop-like swimmer with the typical spatial dimension *l* composed of two counter-rotating shells that share a common axis. For simplicity, we assume that the shells open and close with different angular speeds, *ω*_slow_=*ω* and *ω*_fast_=*pω*, where *ω*_slow_ and *ω*_fast_ stand for the velocity of the slow and fast stroke, respectively, so that *p*≥1 for all swimming gaits. The maximum rotation angle of one plate is *α*/2 and the scallop completes one full cycle in period 
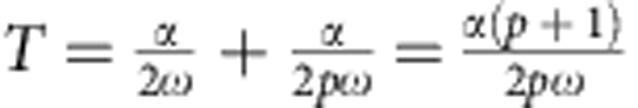
. The momentum transferred to the fluid by the tethered swimmer (or a pump) scales as ~*μ*_app_*Ul*, where *U* is the fluid velocity and *μ*_app_ is an apparent (spatially averaged) viscosity of the fluid, which depends on the instantaneous shear rate, and the history of the flow. For the pivoting shells of the scallop, this leads to the scaling relation ~*μ*_app_*ωl*^2^. Thus, the net force exerted by the pump on the fluid over the stroke period can be estimated (up to an arbitrary function of the opening angle *α*) as


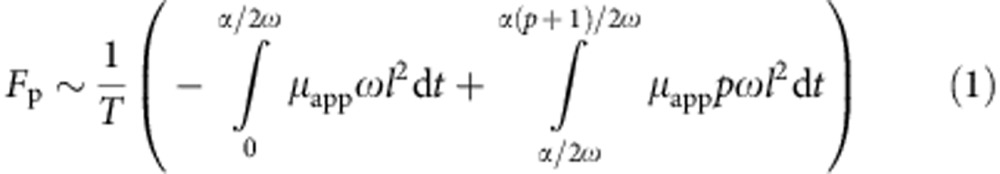


Note that for a Newtonian fluid, the net force over one cycle in equation (1) is zero, as expected from the ‘scallop theorem’, that is, no net momentum can be transferred to a fluid by a pump using geometrically reciprocal strokes^1^. However, for a non-Newtonian fluid, the dependence of the apparent viscosity on the shear rate breaks time–reversibility and from equation (1) the net force over a period is


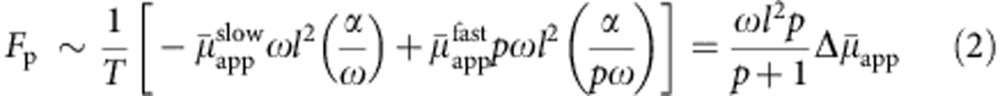


where 
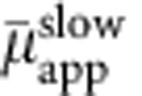
, 
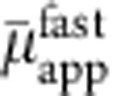
 are the apparent viscosities time-averaged over the slow and fast strokes respectively, with 
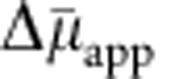
 being their difference.

The propulsion velocity of the force-free swimmer *V*_s_ can be estimated from the dual drag problem, using *F*_p_≈*F*_d_, where *F*_d_ is the force to be applied to an inactive swimmer in order to drag it with velocity *V*_s_^29^. We assume that *F*_d_~μ_*_V_s_*l*, where μ_*_ is some apparent viscosity corresponding to the typical shear rate magnitude *V*_s_/*l*. For low values of the shear rate associated with swimming, that is *V*_s_/*l*≪*ω*, it is reasonable to assume that μ_*_≈*μ*_0_≈const (the rheological measurements (Fig. 4a) suggest that for shear rates up to 1.7 s^−1^ viscosity is roughly constant).

Equating *F*_d_ and *F*_p_ in equation (2) yields the propulsion velocity up to an unknown function of angle *α*:


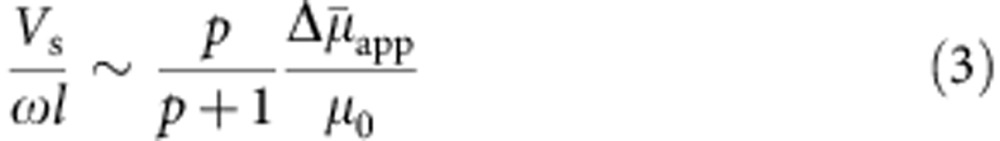


More detailed analysis of a flapping swimmer composed of two infinite plates suggests that the major contribution to 
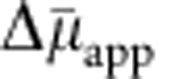
 in equation (3) is because of an abrupt rise (fall) of apparent viscosity of the shear thickening (shear thinning) fluid sandwiched between two close plates at small openings during the fast phase (either closing or opening, see Supplementary Figs 13 and 14).

To model propulsion through shear thickening fluid, a power law equation^30^ was applied to fit the transition region of the viscosity in the shear rate range of 1.5–6 s^−1^ (dotted line in Fig. 4a). The apparent viscosity is 
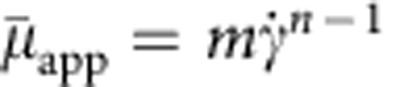
, where *m*, *n* are constants (*m*=0.34, *n*=3.34 as fitted in this case) and 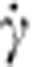 is the shear rate defined as 
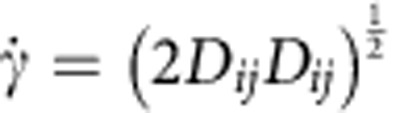
, that is the second invariant of the rate-of-strain tensor 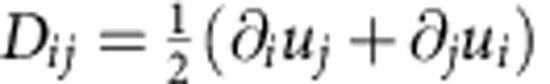. Obviously, in this problem the typical shear rate is, respectively, 
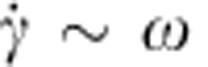
 for opening and 
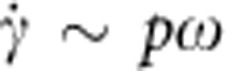
 for closing. Thus, assuming that both (that is, fast and slow) strokes fall within the shear thickening regime, 
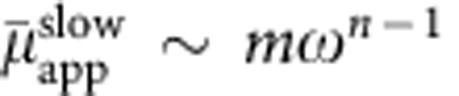
 and 
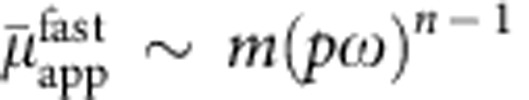
, equation (3) becomes


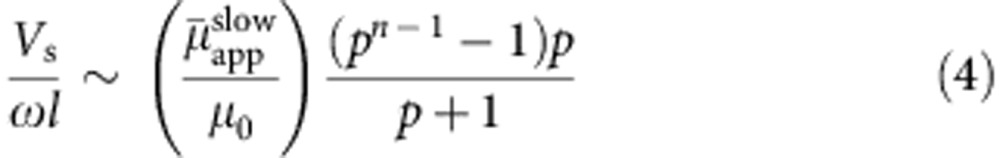


Multiplying *V*_s_ in equation (4) by the stroke period 
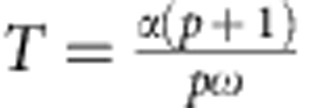
 we obtain a simple expression for the scaled displacement-per-stroke, *D*/*l*,


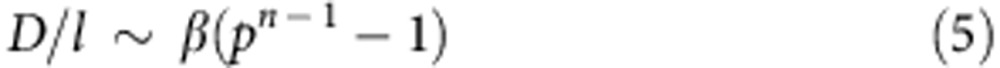


where the sign and magnitude of the dimensionless prefactor *β* depends on the nature of the stroke and fluid properties. The analytical theory therefore clearly confirms that in a shear thickening fluid, the scallop can indeed swim forward using fast closing and slow opening strokes. Alternatively, it can propel in the opposite direction using fast-opening and slow-closing strokes. This is a general result that permits propulsion from symmetric actuation in any of the abundant non-Newtonain fluids, notably those found in biological systems. In order to allow for quantitative comparisons between the predictions of this analytical theory and experiment, we now consider a millimetre-scale low Re scallop-like swimmer actuated by on-board motors. The device, which we call the ‘macro-scallop’ (shown in Fig. 1c and Supplementary Figs 5 and 6), differs from the micro-scallop in the use of rotating hinges, and use of motors, which permit rapid and precisely controlled opening and closing of the swimmer’s shells. It has a characteristic length ~15 mm, an average linear velocity ~25 mm s^−1^ during the fast closing stroke and 6.25 mm s^−1^ during the slow opening stroke, so that the Re=0.017 for closing and Re=0.094 for opening, which are still low enough to neglect the inertia of the swimmer.

Tests of the analytical theory were conducted in shear thickening fluid in two regimes: (i) fixed opening speed *ω*_o_ over a range of closing speeds *ω*_c_>*ω*_o_ (Fig. 4b) and (ii) fixed closing speed *ω*_c_ at different opening speeds *ω*_o_>*ω*_c_ (Fig. 4c). When the closing speeds (solid squares) are larger than the opening speed (hollow triangle), the net displacement *D*/*l* is larger than 0 and the macro-scallop moves in the forward direction (hinge leading). On the other hand, when the opening speeds (hollow triangles) are larger than the closing speed (solid square), the swimmer moves in the opposite direction with a negative net displacement *D*/*l*<0 (hinge trailing). Both experimental results are in excellent agreement with the theory of equation (5) based on scaling arguments. It follows that a symmetric scallop can indeed move, provided time-reversal symmetry is broken by the asymmetric actuation speeds of opening and closing in a non-Newtonian fluid. Note that the two actuation gaits (that is, fast opening/slow closing versus fast closing/slow opening) result in a different displacement for the same value of *p* because of viscosity hysteresis (see Supplementary Note 3 and Supplementary Fig. 10).

### Numerical model of propulsion

**Figure 5:**
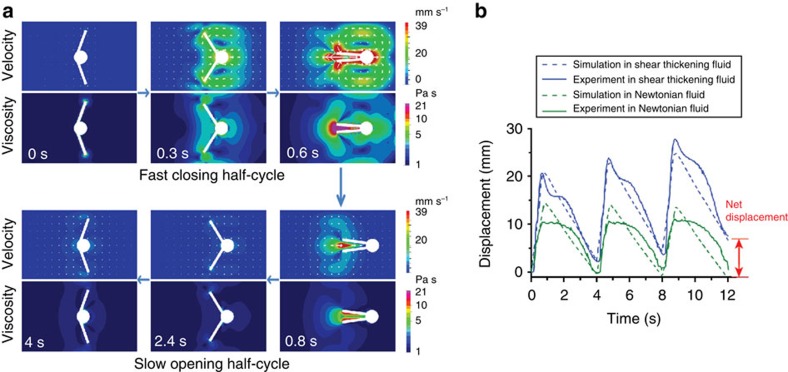
Numerical simulation of propulsion by reciprocal motion in a shear thickening fluid. (**a**) Fluid velocity and viscosity fields around the swimmer in shear thickening fluid (see also Supplementary Movie 4 and Supplementary Fig. 15 for enlarged images). The three images in the upper panel correspond to the fast closing half-cycle (~0.8 s) and the images in the lower panel correspond to the slow opening half-cycle (~3.2 s). The simulation result verifies that the net displacement is a result of the viscosity differences during the two half-cycles, which is caused by the differences in fluid shear rate (velocity gradient) under asymmetric actuation. (**b**) The displacement curves of the asymmetric actuated macro-scallop in shear thickening (blue) and Newtonian fluid (green) for three cycles. Simulation results (dashed lines) are consistent with experimental data (solid lines), where the macro-scallop exhibits net displacement in the shear thickening fluid but no net displacement in the Newtonian fluid.

We also employ numerical simulations to study the propulsion mechanism of reciprocal motion in non-Newtonian fluids using the open-source Computational Fluid Dynamics (CFD) package FeatFlow (detailed method in the Supplementary Note 1). Figure 5a and Supplementary Movie 4 show the fluid velocity and viscosity fields at six frames through the complete 4-s propulsion cycle of the macro-scallop swimming in a shear thickening fluid. The net displacement in one cycle is clear from the difference in swimmer position between the start (0 s) and finish (4 s) of the cycle. The propulsion mechanism is illustrated by comparing the field maps at 0.3 and 2.4 s; these frames both correspond to the same *α*=115°; however, the higher closing angular velocity of the shells leads to a much higher velocity gradient (shear rate) of the fluid at 0.3 s, which results in larger viscosity between the two shells relative to that during the opening stroke (at 2.4 s). Therefore, the forward displacement during the fast closing half-cycle (upper panel in Fig. 5a) is larger than the backward displacement in the slow opening half-cycle (lower panel in Fig. 5a), which leads to the net displacement over one period. The displacement curves (Fig. 5b) of the simulation show excellent quantitative agreement with the experimental data in both the Newtonian and shear thickening cases. The simulation results demonstrate that the net propulsion is a result of the viscosity differences during the two half-cycles, which is caused by differential apparent fluid viscosity under asymmetric shearing conditions. Importantly, consistent results were achieved using only the simple shear thickening relationship between fluid viscosity and instantaneous shear rate. This suggests that the dominant factor leading to net propulsion in our experiment is differential viscosity rather than fluid energy storage mechanisms such as fluid elasticity or viscosity hysteresis (thixotropy/rheopecty).

## Discussion

We show that low-Re propulsion of a scallop is possible in shear thickening and shear thinning fluids. The devices are true swimmers, propelled by periodic body-shape changes.^19^ Differences in the opening and closing rates give rise to differences in the corresponding shear rates and hence the viscosities of the non-Newtonian fluid. The result is net propulsion despite the reciprocal stroke. Unlike ref. 27, fluid elasticity plays only a minor role; rheological measurements (Supplementary Figs 7 and 8) show that the first normal stress difference of our shear thickening medium is two orders of magnitude smaller than that of the Boger fluid used in ref. 27. Furthermore, the numerical simulations, which do not include elasticity effects, accurately reproduce the motion, and experiments with a time-symmetric stroke (*p*=1) produce no net propulsion. In summary, our swimmer fulfills three conditions necessary for propulsion by reciprocal motion at low Re in the absence of elasticity: (1) absence of mirror symmetry in the direction of motion (that is, a clear fore-aft asymmetry), (2) time-asymmetric actuation and (3) the coupling of such an actuation to a non-Newtonian fluid rheology. As the time asymmetry of the stroke is responsible for the net displacement, the direction of propulsion can be reversed by inverting the opening and closing speeds. This is a potential benefit over other micropropulsion schemes where the direction of motion is dictated by the fixed spatial asymmetry of the device^18^,^27^.

We found that the average velocity of the micro-scallop in the shear thickening fluid is faster than that in the shear thinning one, as for the same difference in shear rates the change in fluid viscosity is larger in the shear thickening fluid. This is apparent from the differences in the *n* exponents in the power law models. The swimming velocity can be increased by maximizing the difference in the opening and closing speeds, up to the point where the viscosity plateaus at ~7 s^−1^. Optimizing the shape of the swimmer can also be expected to improve performance. The viscosity is highly dependent on the shear rate, which is not only determined by the speed of opening and closing but is also a function of the swimmer shape. Optimizing the geometric shape of the swimmer (and the morphology of its surfaces) may thus have a significant effect on propulsion speed of a non-Newtonian microswimmer.

A simple theory that relies on scaling arguments is derived that captures the essential underlying physical mechanism of the locomotion, that is, via shear rate modulation of the viscosity. The highest shear rate is achieved when the fluid is sandwiched between the two shells at small openings during the fast stroke phase and the corresponding abrupt change of the apparent viscosity controls the net displacement over a stroke. The weakly thixotropic property of the shear thickening fluid also plays a minor role in the propulsion. In our case, the viscosity hysteresis provides a speed advantage to those gaits where the *ω*_o_>*ω*_c_ versus those where *ω*_c_>*ω*_o_. In principle, large viscosity hysteresis could be used to generate net displacement even for time-symmetric strokes.

Microswimmers have the potential to be useful in biomedical applications or as rheological probes *in vivo*. Swimming in biological fluids is a first step to achieve these goals. Hyaluronic acid is found in connective, epithelial and neural tissues^31^; moreover, it is one of the main components of the extracellular matrix, which contributes significantly to cell proliferation and migration^32^. Many biological media including saliva, blood, vitreous and synovial fluid exhibit shear thinning properties, and as we show here this can be exploited in the design and operation of a microswimmer that is simpler to operate than many other microrobots. From an engineering point of view, reciprocal motion can be achieved with much simpler actuation schemes compared with nonreciprocal actuators. Most existing (miniaturized) actuators are reciprocal, including piezoelectric, bimetal stripes, shape memory alloy, heat- or light-actuated polymers, which can all potentially be used as actuators for the microswimmer demonstrated here. Thus, the reciprocal swimming mechanism of the micro-scallop reported in this paper may provide a general scheme for micro-swimming in biological fluids.

## Methods

### Micro-scallop design and fabrication process

The micro-scallop was readily realized by 3D printing and micro-molding technique, shown in Supplementary Fig. 1. Supplementary Fig. 2 shows the detailed dimensions of the micro-scallop. The hinge is both narrow (200 μm) and thin (60 μm) to decrease the elastic force against the magnetic force, while the two shells are much thicker (300 μm) to avoid any deformation during the actuation. This ensures that the motion is reciprocal, as stated by Purcell that any single-hinge structure can only result in a reciprocal motion. The shells (800 μm) are also wider than the hinge (200 μm), which enables larger contact area with the fluid and results in better propulsion.

The negative mold of the micro-scallop (shown in Supplementary Fig. 1) was printed with a high-temperature material (RGD 525) using a 3D printer (Objet260 Connex, Stratasys, Israel). The support-material was removed by magnetic stirring in KOH solution (0.03 g ml^−1^) for 12 h. Two grams of Ultra Green V10 Glow powder (2–8 μm, Glow Inc., MD) was mixed with 15 ml ethanol by sonication for 1 h, and the supernatant was collected and dried under vacuum. Two grams base agent of PDMS (Sylgard 184, Dow Corning) was added to the dried Glow powder, mixed thoroughly under sonication for 1 h. A curing agent (100 μl) was then added, and the solution was thoroughly mixed and degassed for 1 h. The 3D printed mold was filled with the prepared PDMS solution and degassed for 0.5 h and cured at 65 °C for 1.5 h. Finally, the PDMS shell was released from the mold and two neodymium micromagnets (Ø0.2 mm × 0.4 mm, GMB Magnete Bitterfeld GmbH, Germany) were attached to the shells using cyanoacrylate (Ultra Gel, Pattex) in two steps. As illustrated in Supplementary Fig. 3a, one macro-magnet was first attached on the left side and the micro-magnet on the left was glued to the PDMS at the correct orientation under the stereoscope. After the fixation of the first micro-magnet, the macro-magnet was moved to the right, and the second micro-magnet on the right was glued to the PDMS (Supplementary Fig. 3b). During the second step, the local field dominated by the field of the macro-magnet; therefore, the orientation of the second micro-magnet was kept in the opposite direction of the first one.

### Preparation of the fluids and rheological measurements

Fumed silica suspensions (8% w/w) were used as the shear thickening fluid^28^. Fumed silica powder (Aerosil 150, Evonik, Germany) was mixed thoroughly with poly(propylene glycol) (PPG, *M*_w_=725, Sigma-Aldrich). The solution was then degassed for 3 h.

Hyaluronic acid (6 mg ml^−1^) was used as the shear thinning fluid. Hyaluronic acid powder (53747, Sigma-aldrich) was mixed in PBS (Gibco, Life Technologies) and stirred under room temperature for 48 h.

Glycerol (99.5%, 1,410 mPa s at 20 °C, VWR, France) and silicone oil (Dow Corning 200/12,500 cSt, VWR, UK) were used as Newtonian fluids for the micro-scallop and macro-scallop, respectively.

The viscosities were measured using shear rate ramp experiments on a rheometer (Kinexus Pro, Malven, UK) using a plate-to-plate (40 mm in diameter) set-up. The temperature was set to 25 °C. The viscosity was measured in the range of 0.05–300 s^−1^ with a 4-min ramp time. The data shown in Fig. 5 and Supplementary Fig. 9 are the average of three independent measurements of two fluids, respectively.

### Actuation set-up and video analysis of the micro-scallop

The micro-scallop was actuated by an external magnetic field, generated by a Helmholtz coil that can produce homogeneous magnetic fields of up to ~300 G in the swimming direction of the micro-scallop (indicated by the red arrow in Fig. 2a). As the micromagnets have much higher density (~7,400 kg m^−3^) than the fluids (~1,000 kg m^−3^), it will sink to the bottom in an upright container and then the friction between the micro-scallop and the bottom will severely affect the propulsion of the micro-scallop. Therefore, the fluidic channel was placed in a reversed manner to minimize the friction, and the micro-scallop was held on the interface of fluid and air by interfacial force, while the micro-scallop was immersed in the fluid, as illustrated in Supplementary Fig. 4. The width and height of the fluidic channel are 5 and 3 mm, which are about eight and four times of the corresponding size of the micro-scallop to minimize the boundary effect of swimming at low Re.

Videos of the micro-scallop were taken under a stereoscope (MZ95, Leica, Germany) with a CCD camera (DFC490, Leica) at 20 frames per s, with ultraviolet illumination by a LED (peak wavelength at 365 nm, Roithner Laser Technik GmbH, Germany) and a coloured-glass filter (485–565 nm, VG9, Schott, Germany). Every frame of the video was extracted using ffmpeg and the sequential images were analysed using customized Matlab script to extract the coordinates of the midpoint of the hinge and the angle between the two shells in every frame. The frames, in which the angle *α* reaches its minimum value of 40°, are selected from each period and are used to deduce the displacement. Five selected frames between 0 and 200 s with interval of 50 s were overlapped in Fig. 3a,b using ImageJ (NIH), and for comparison the micro-scallop was aligned at the same *y* axis position. Each experiment was repeated five times.

### Macro-scallop design and experimental set-up

Supplementary Fig. 5 is the 3D model of the macro-scallop model swimmer. It was designed to be the smallest size possible for a low Re using commercially available motors. Two brushless DC-motors (0308H with a 03A gear head, total size 3.4 mm in diameter and 12.6 mm in length, Faulhaber, Germany) were used, and the speed and the rotational direction are controlled by a controller (SC1801F, Faulhaber) with analogue input 0.5–12 V to set the speed value. One full period of the macro-scallop was set to 4 s, with ~0.8 s for fast closing half-cycle and 3.2 s for slow opening half-cycle (Fig. 5a and Supplementary Fig. 6b).

The shells are made of carbon fibre sheet (0.3-mm thick, Conrad, Germany). Two sizes were used for the experiment, that is, 16 mm × 14 mm (length × width) for the illustration of swimming comparison with the numerical simulation results; and 8 mm × 7 mm (length × width) to test the theory of the scaling argument. Small area shells decrease the power needed for rotation in very viscous fluid; thus, the motors can provide a higher rotating velocity and achieve a higher ratio *p* of closing and opening angular velocities.

As illustrated in Supplementary Fig. 6a, a linear air track (Edu-lab, UK) was used to balance the weight of the macro-scallop. For imaging, green-phosphorescent tape (Conrad) was stuck to the bottom of the shells and the body. A ultraviolet lamp (peak wavelength at 365 nm) illuminated from the side of the tank and the video was taken by a digital camera at 25 frames per s with a coloured-glass filter (485–565 nm, VG9, Schott). Supplementary Fig. 6b is a series of time-lapse pictures showing one cycle of slow-open–fast-close asymmetric actuation of the macro-scallop and the resulted net displacement in shear thickening fluid (also see Supplementary Movie 6), while there was no net displacement by symmetric actuation in the same fluid (see Supplementary Movie 7).

### Test of the theory by scaling argument

The macro-scallop with shells of 8 mm × 7 mm (length × width) was used to test the theory of the scaling argument. The closing half-cycle and the opening half-cycle were tested separately at various angular velocities. The largest opening angle *α*=295° and the smallest closing angle *α*=10° were kept the same for all the tests by mechanical limits. Each velocity measurement was repeated at least three times.

The time of shell closing or opening and the resultant displacements were calculated from the recorded video. Every frame of the video was extracted with ffmpeg. The first frame in which the swimmer moved was labelled as the start frame. The last frame in which the swimmer stopped moving was labelled as the end frame. In the start and end frames, the frame number *N*_1_ and *N*_2_, *x* and *y* coordinates of the body tip (*x*_1_, *y*_1_) and (*x*_2_, *y*_2_), were measured in ImageJ (NIH), respectively, so that the following parameters were calculated:

Time of the half-cycle, 
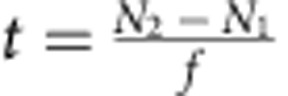
, where *f*=25 frames per s is the frame rate;

Average angular velocity, 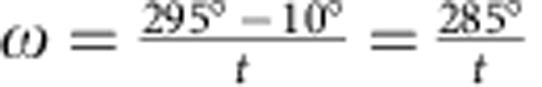;

Displacement over half-cycle, 
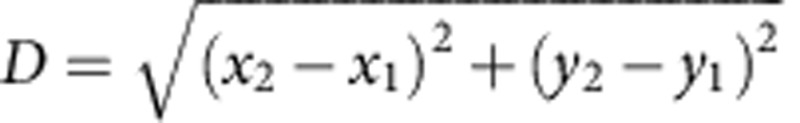
 for the forward displacement in the closing half-cycle, and 
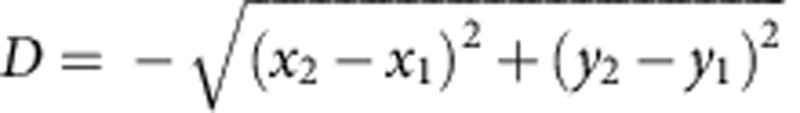
 for the backward displacement in the opening half-cycle.

The scaled displacement-per-stroke, *D*/*l*, were calculated, where the characteristic length of the micro-scallop *l*=7 mm, and it is plotted against the ratio *p* of average angular velocities in Fig. 4b,c to compare with the scaling theory.

### Numerical simulation and analytical analysis

The numerical simulations were conducted using the CFD package FeatFlow (www.featflow.de) as a solver for the incompressible Navier-Stokes equations. A quasi 2D approach was used in such a way that the thickness of the computational domain was set to a small value. The rheological properties of the non-Newtonian fluid were modelled by a piecewise continuous approximation to the measured viscosity profile (Supplementary Fig. 12). The simulations were conducted over three complete opening/closing cycles. The simulation volume was 45 mm × 90 mm × 1 mm, using 60,000 timesteps with d*t*=0.0002, s. Meshes were generated by a grid-adaptation technique that concentrates vertices at the fluid–solid interface (Supplementary Fig. 11). The methods for the numerical simulation and analytical analysis are discussed in more detail in the Supplementary Notes 1 and 2, respectively.

## Author contributions

T.Q., T.-C.L., A.G.M. and P.F. designed the experiments. T.Q. performed the experiments. T.Q., T.-C.L. and A.G.M. analysed data. K.I.M. and A.M.L. developed the analytical theory. R.M., O.M. and S.T. performed numerical simulations. P.F. conceived the project and supervised the experiments. All authors contributed to the writing of the paper.

## Additional information

**How to cite this article**: Qiu, T. *et al*. Swimming by reciprocal motion at low reynolds number. *Nat. Commun.* 5:5119 doi: 10.1038/ncomms6119 (2014).

## Supplementary Material

Supplementary Figures, Supplementary Notes and Supplementary References Supplementary Figures 1-16, Supplementary Notes 1-3 and Supplementary References

Supplementary Movie 1 Micro-scallop in shear thickening fluid. Net displacement in x direction was achieved by asymmetric actuation of slow-open-fast-close (upper panel), in comparison, no net displacement in x direction by symmetric actuation (lower panel). The movie was sped up 4-times.

Supplementary Movie 2 Micro-scallop in shear thinning fluid. Net displacement in x direction was achieved by asymmetric actuation of fastopen-slow-close (upper panel), in comparison, no net displacement in x direction by symmetric actuation (lower panel). The movie was sped up 4-times.

Supplementary Movie 3 Micro-scallop in Newtonian fluid. An asymmetric actuation of slow-open-fast-close was applied to the micro-scallop and no net displacement in x direction was observed. The movie was sped up 4-times.

Supplementary Movie 4 Numerical simulation results of the velocity fields around the macro-scallop under asymmetric slow-open-fast-close actuation. Upper panel: the swimmer in Newtonian fluid results in no net displacement; lower panel: the swimmer in shear thickening fluid results in net displacement.

Supplementary Movie 5 Numerical simulation shows the velocity (upper panel) and viscosity fields (lower panel) of the shear thickening fluid around the macro-scallop under asymmetric slow-open-fast-close actuation.

Supplementary Movie 6 Experimental video shows asymmetric slow-open-fast-close actuation of the macro-scallop resulted in net displacement in shear thickening fluid. The movie was sped up 2-times.

Supplementary Movie 7 Experimental video shows symmetric actuation of the macro-scallop resulted in no net displacement in shear thickening fluid. The movie was sped up 2-times.

Supplementary Movie 8 Experimental video shows asymmetric slow-open-fast-close actuation of the macro-scallop resulted in no net displacement in a Newtonian fluid. The movie was sped up 2-times.

Supplementary Movie 9 Micro-scallop actuation in the bulk of a shear thickening fluid. It is immersed in the fluid, far away from meniscus and all walls, using the setup shown in Supplementary Fig. 16a. The scallop falls under gravity throughout the experiment, but with a time-asymmetric stroke it clearly propels in the transverse direction (left panel), in comparison, no net displacement in x direction under symmetric actuation (middle panel) or no actuation (right channel). The movie was sped up 4-times.
