# Supplementary material for: Swimming by reciprocal motion at low Reynolds number
Source: Nat Commun. 2014 Nov 4;5:5119. doi: 10.1038/ncomms6119 (PMC4241991; doi:10.1038/ncomms6119)
Supplement: Supplementary Figures, Supplementary Notes and Supplementary References — Supplementary Figures 1-16, Supplementary Notes 1-3 and Supplementary References [file ncomms6119-s1.pdf]

Supplementary Figures

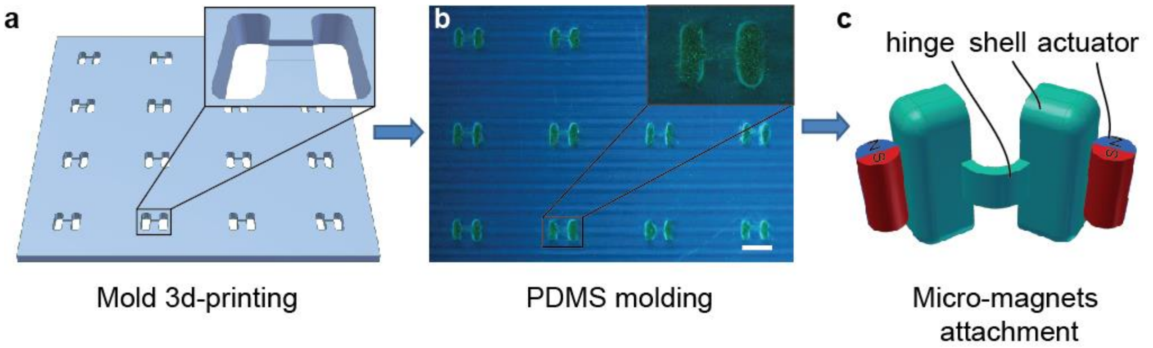

Supplementary Fig. 1. Fabrication process of the micro-scallop. (a) The negative mold of the micro-scallop is made by 3-D printing. The mold for the hinge is much shallower and narrower than the two shells. Shown in the inset is an enlargement of one mold. (b) As-printed molds filled with PDMS polymer containing green fluorescent powder imaged under UV light (scale bar is 1 mm). (c) 3-D model of the resultant micro-scallop. The PDMS part (green) is released after curing and two micro-magnets are attached in the orientation indicated in the schematic. A more detailed description of the fabrication procedure is illustrated in Supplementary Fig. 3.

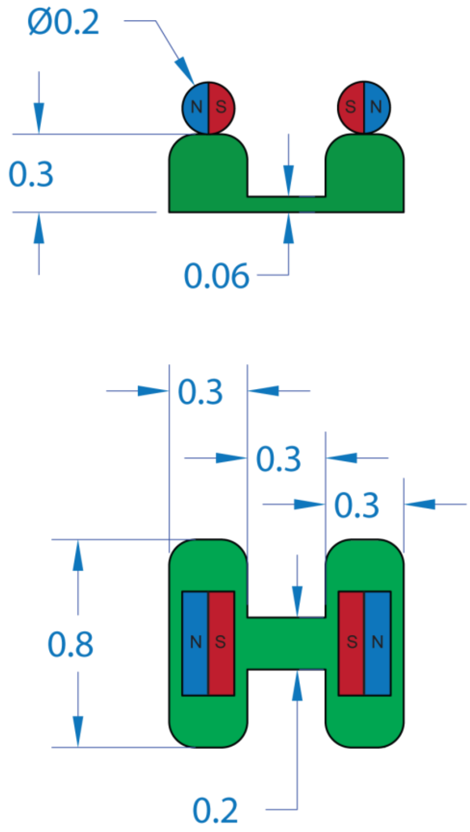

Supplementary Fig. 2. Detailed dimensions of the micro-scallop. Important dimensions of the micro-scallop are drawn in the figure in the unit of millimeter.

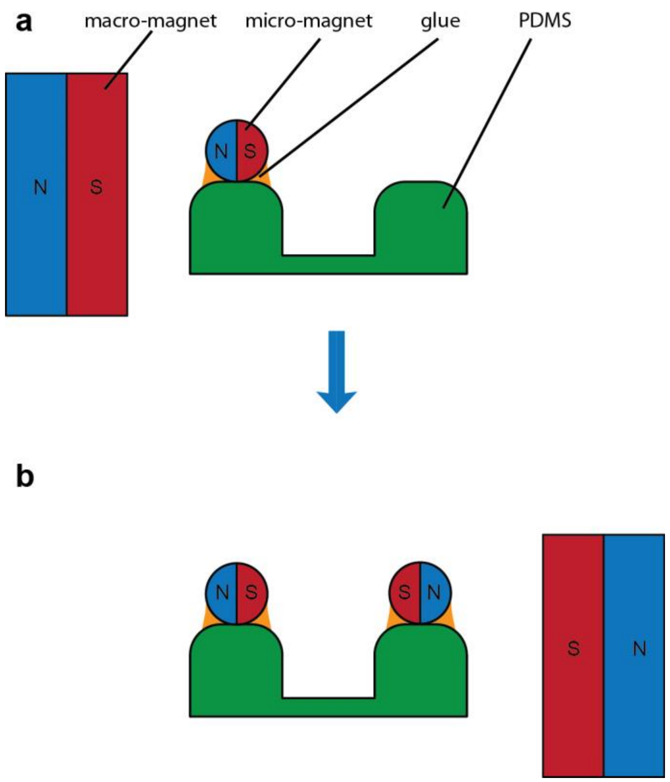

Supplementary Fig. 3. Illustration of the micro-magnets attachment process. (a) Step 1, the first micro-magnet (left) is attached to the PDMS by glue, while its orientation is kept by the macro-magnet. (b) Step 2, after the fixation of the first magnet, the second micro-magnet (right) is attached to the PDMS by glue. The local field is dominated by the macro-magnet on the right, so the orientation of the second micro-magnet is kept in the opposite direction of the first. The macro-magnet is not drawn to scale.

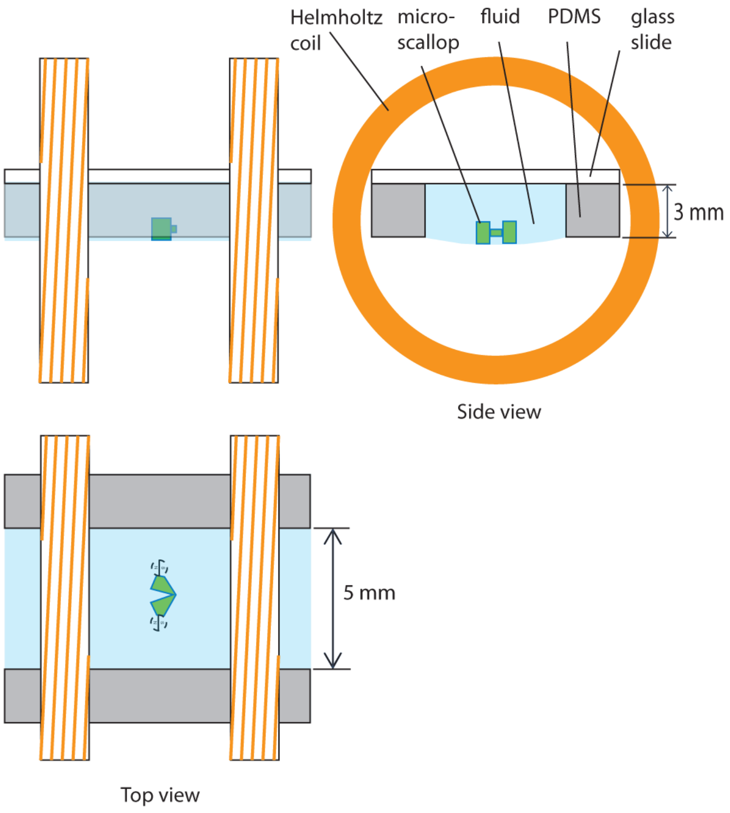

Supplementary Fig. 4. The actuation setup for the micro-scallop. The micro-scallop is not drawn to scale.

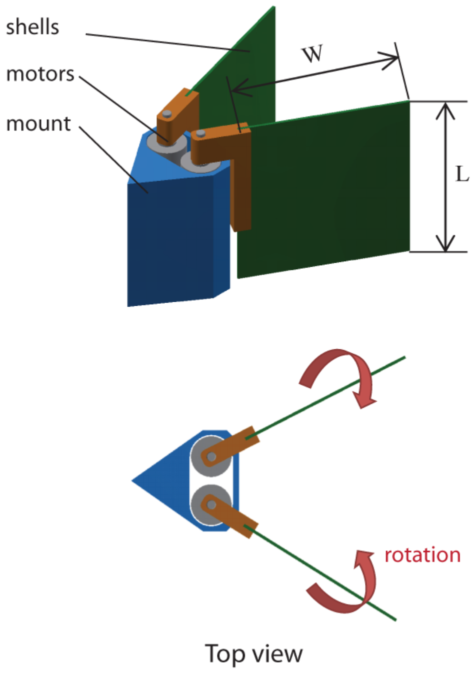

Supplementary Fig. 5. Macro-scallop 3-D model and its top view illustrating the closing motion of the two shells.

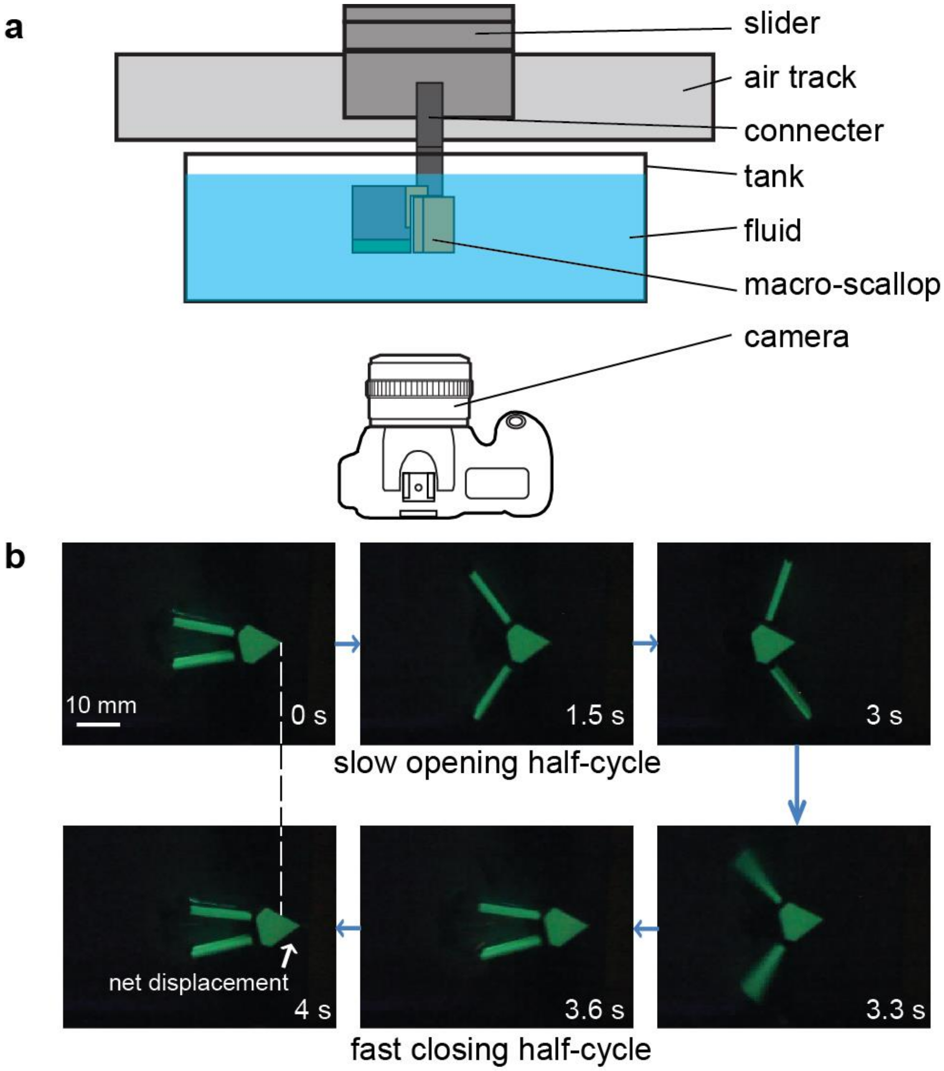

Supplementary Fig. 6. Propulsion experiments of the macro-scallop. (a) An illustration of the experimental setup for the macro-scallop. (b) Time-lapse pictures of asymmetric actuation of the macro-scallop in shear thickening fluid. The three pictures in the first line correspond to the slow opening half-cycle (~3 s) and the pictures in the second line correspond to the fast closing half-cycle (<1 s). The net displacement is observed by comparing the pictures at 0 s and 4 s with the dashed line aligned with the tip of the macro-scallop at 0 s.

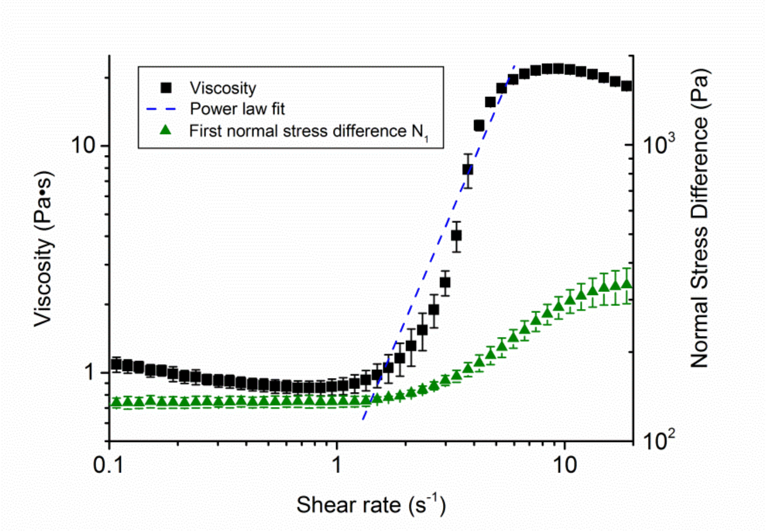

Supplementary Fig. 7. Apparent dynamic viscosity of the shear thickening fluid. Power law model (dotted line) is used to fit the viscosity (black squares) in the shear rate range of 1.5~6 s^{-1}. The change of first normal stress difference N_1 of our shear thickening medium is two orders of magnitude smaller than the Boger fluid used in [8]. Thus, the viscosity change is dominant during the swimming process. The error bars represent standard deviations.

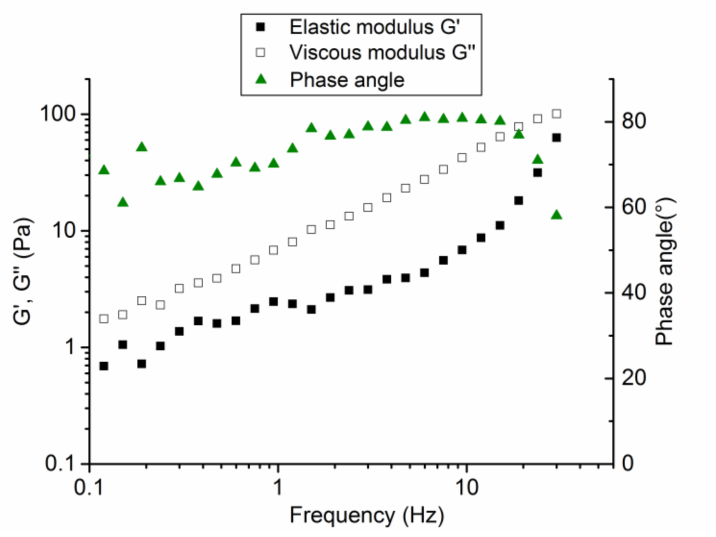

Supplementary Fig. 8. Oscillation test of the shear thickening fluid. The viscous modulus G’’ is more than 2 times larger than the elastic modulus G’ over the frequency range of 0.1~20 Hz, and the phase angle is 70º ~80º, which both indicate that the viscosity is dominant over elasticity for the shear thickening fluid.

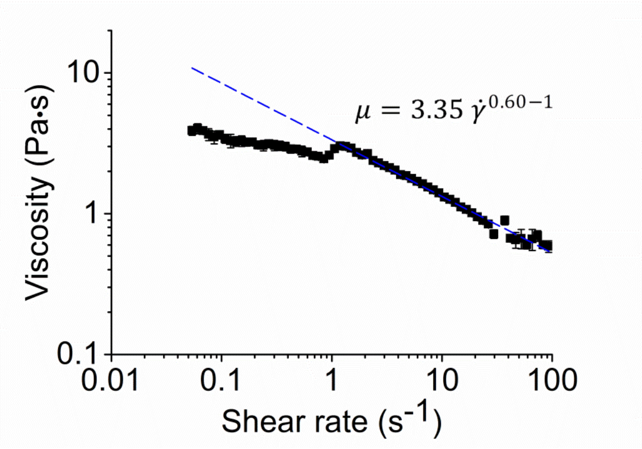

Supplementary Fig. 9. Apparent dynamic viscosity of the shear thinning fluid. Power law model (dotted line) is used to fit the data in the shear rate range of 1~100 s^{-1}. The error bars represent standard deviations.

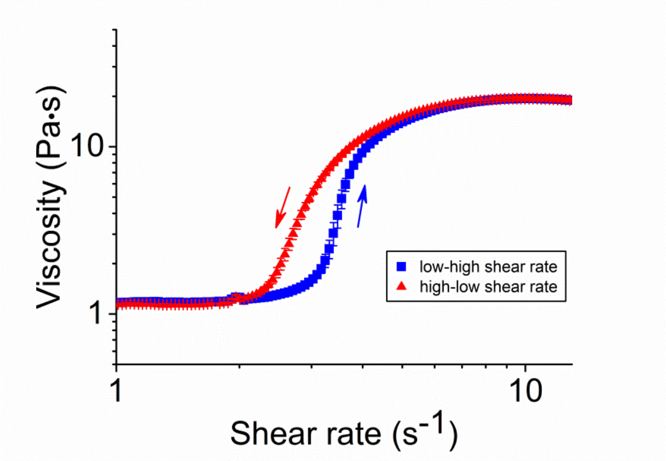

Supplementary Fig. 10. Hysteresis of viscosity of the shear thickening fluid. When the shear rate increases from a low viscosity (blue squares), the transition occurs at a higher critical shear rate, while when the shear rate decreases from a high viscosity (red triangles), the transition takes place at a lower shear rate. The error bars represent standard deviations.

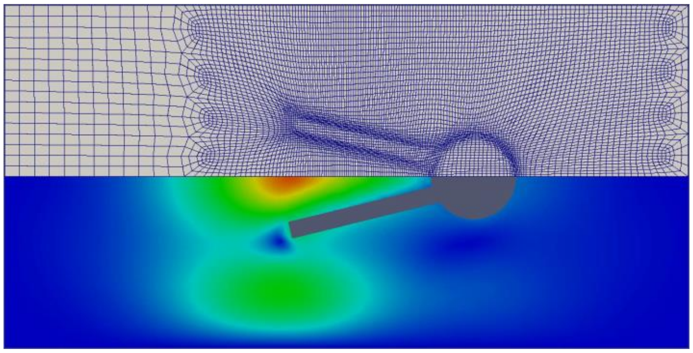

Supplementary Fig. 11. The vertices of the computational mesh are concentrated near the surface of the macro-scallop to improve resolution of the liquid-solid interface. The color in the lower half shows the velocity field of the fluid.

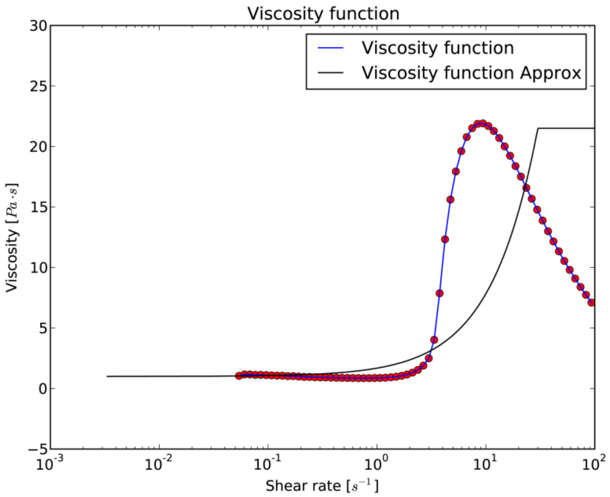

Supplementary Fig. 12. Approximation of the viscosity for shear thickening fluid for convergence in numerical simulation. Red circles are measured data points in experiment, blue line is the trend line, and the black line is the approximation used in numerical simulation.

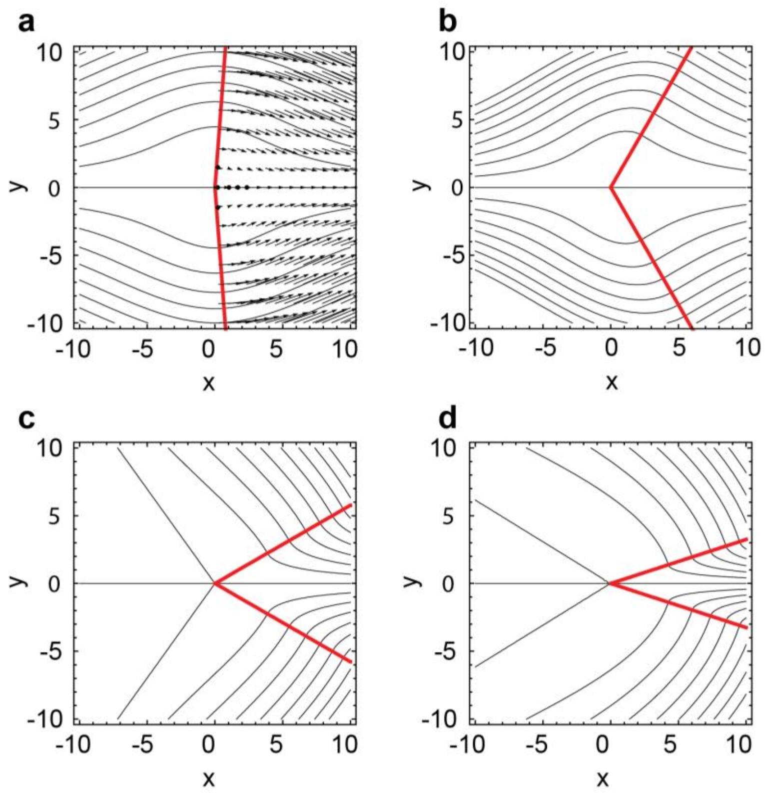

Supplementary Fig. 13. Flow pattern (streamlines) due to a 2D tethered infinite “scallop” (pump) upon varying the opening α=85.7°(a), 60°(b), 30°(c) and 18°(d); position of the plates are marked by thick red lines. The corresponding vector velocity field is in (*a*) for the closing stroke.

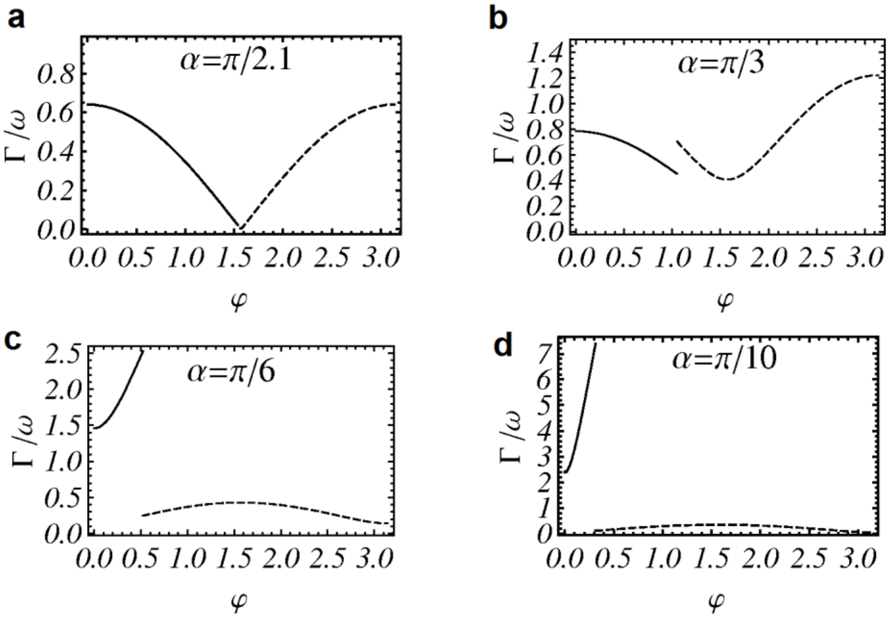

Supplementary Fig. 14. The scaled strain rate Γ/ω for the 2D infinite tethered "scallop" (pump) *vs.* a polar angle φ for the same openings α as in Supplementary Fig. 13 (shown in radians). Solid lines stand for the inner region and the dashed lines stand for the outer region.

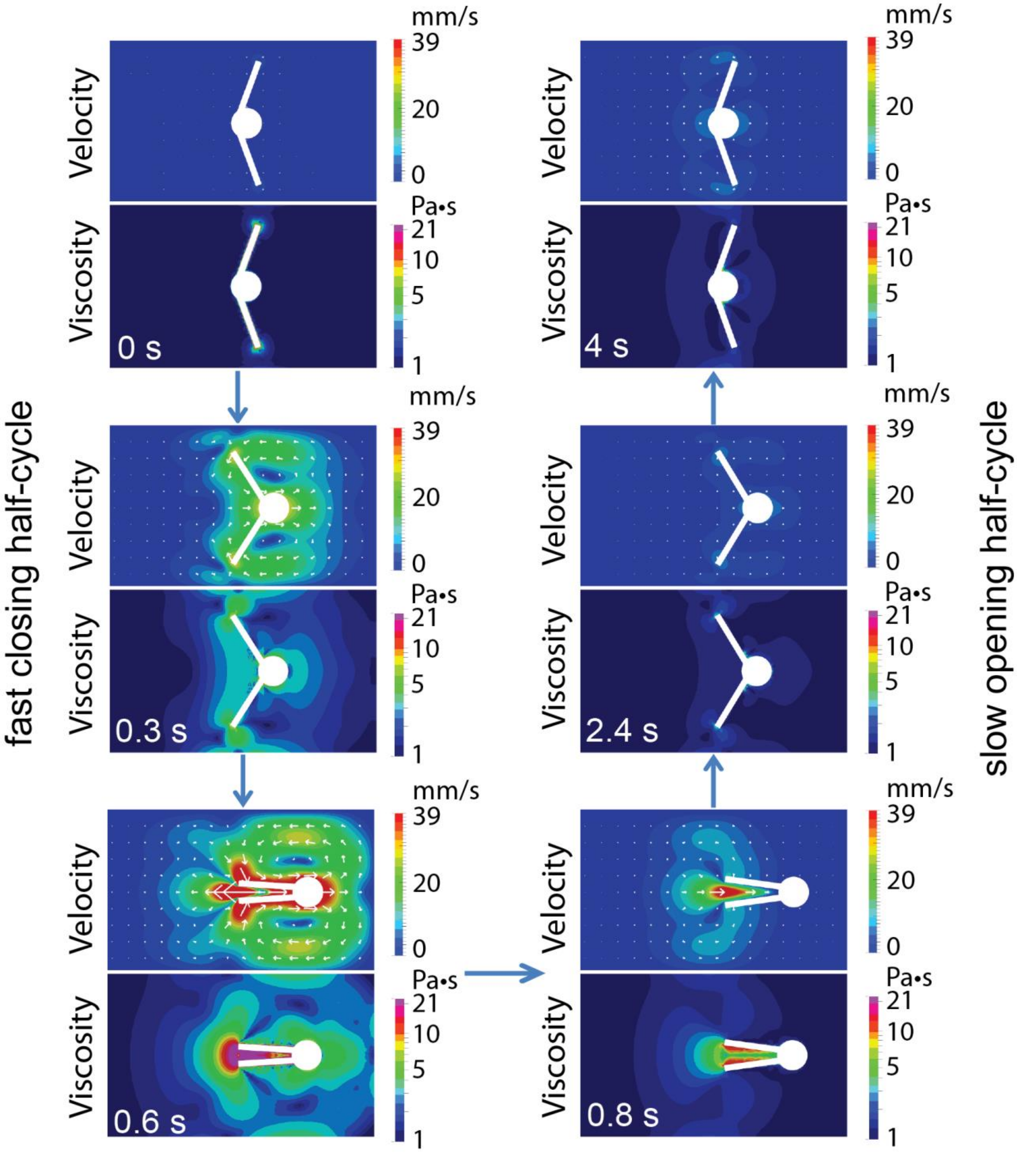

Supplementary Fig. 15. Numerical simulation of propulsion by reciprocal motion in a shear thickening fluid. Enlarged images of fluid velocity and viscosity fields around the swimmer in shear thickening fluid.

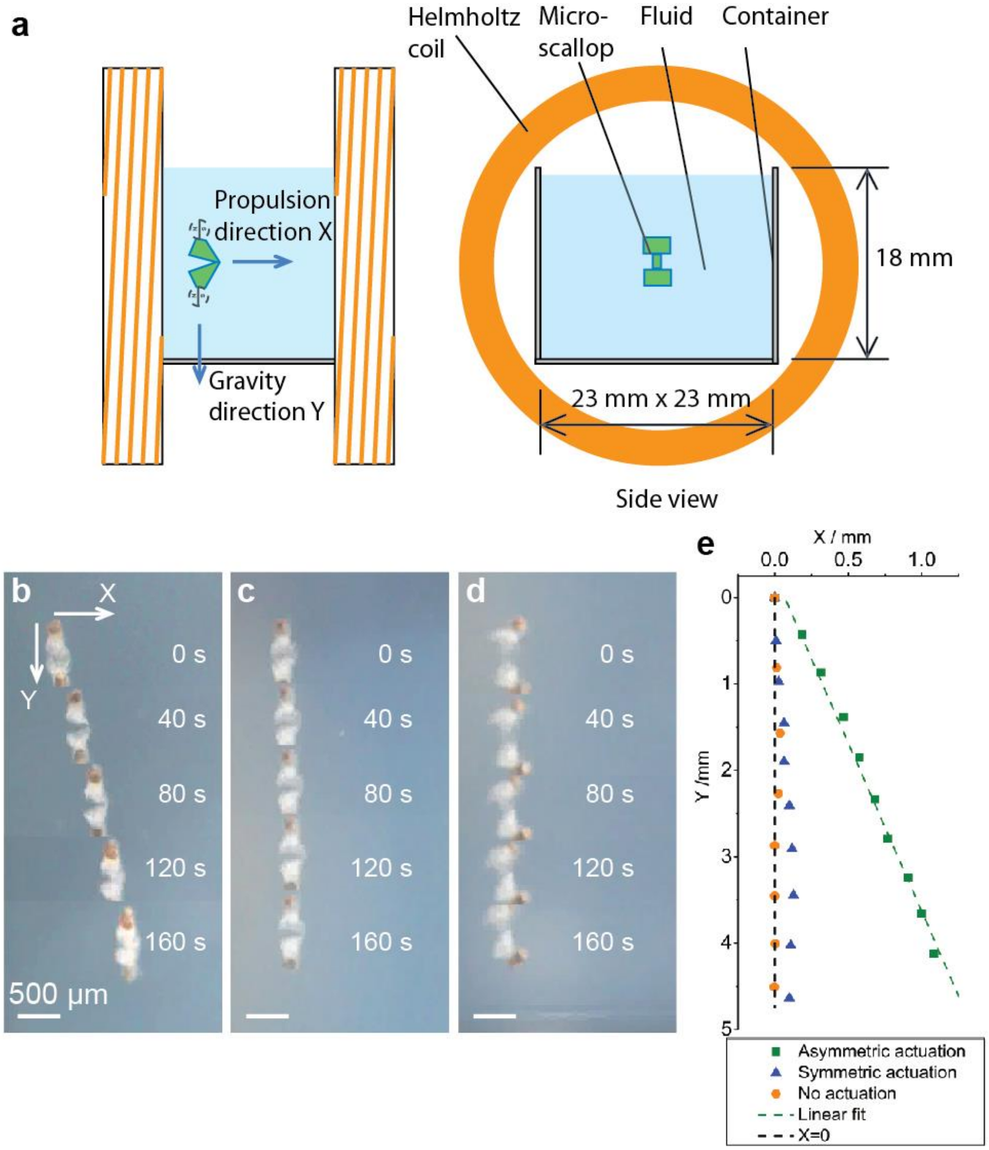

Supplementary Fig. 16. Interface-free Micro-scallop swimming test in shear thickening fluid while falling under gravity. (a) Schematic showing the actuation setup for the micro-scallop, which is not drawn to scale. The micro-scallop is immersed in the fluid, far away from the meniscus and all walls. The propulsion is in the X direction, which is independent from the falling motion caused by gravity in the Y direction. The video is taken by camera from the side. (b)-(d) Time lapse composite-pictures of 5 frames at an interval of 40 s (10 strokes). The Y positions of the micro-scallop in the three pictures are aligned at 0 s for comparison. (b) Asymmetric actuation results in net propulsion in X direction while the swimmer falls in Y direction (see movie S upper panel). (c) Symmetric actuation results in no net propulsion in X direction (see Supplementary Movie 9 middle panel). (d) No actuation of the swimmer results in a vertical falling in the direction of gravity. A very weak (~1.5 G) homogeneous magnetic field is used to keep the swimmer in fixed orientation while falling. (e) The displacement plot of the three tests in (b)-(d). The time interval between dots is 20 s (5 strokes). The swimmer has a propulsion speed of 5.2±1.4 µm/s under asymmetric actuation, which is similar to the results observed when the swimmer is suspended by the air-liquid meniscus. In cases of symmetric actuation or no actuation, the swimmer falls vertically and shows no net displacement in X direction.

Supplementary Notes

Supplementary Note 1. Numerical simulation of the macro-scallop

The numerical simulations of the macro-scallop in non-Newtonian and Newtonian fluids were conducted using the open-source CFD (Computational Fluid Dynamics) package FeatFlow (www.featflow.de). We configured the 3D simulation to use a pseudo 2D setup which means that the thickness of the swimmer and the computational domain are reduced. Thus the number of degrees of freedom in the simulation is significantly reduced as is the computational cost. In the following we will briefly explain the numerical methods used to simulate the macro-scallop swimmers in Newtonian and non-Newtonian fluids. In our FeatFlow software the fluid is modelled by the incompressible Navier-Stokes equations which can be formulated as

$$\begin{cases} \varrho\partial_t\vec{v} + \varrho(\vec{v}\cdot\nabla)\vec{v} + \nabla p = \nabla\cdot\mathbb{S} + f \\ \nabla\cdot\vec{v} = 0 \end{cases}$$ (Supplementary Eq. 1)

$$\mathbb{S} = \mu(\nabla\vec{v} + \nabla\vec{v}^T)$$ (Supplementary Eq. 2)

where we denote the constant density by ρ, the shear dependent viscosity by μ, the unknown velocity and pressure by the pair $(\vec{v}, p)$, and the viscous stress tensor by $\mathbb{S}_{ij} = 2\mu D_{ij}$, in which D_{ij} is the rate-of-strain tensor.

This system of equations is discretized using the Finite Element Method (FEM) which is implemented in our FeatFlow CFD package together with the recent extensions of non-Newtonian fluids and dynamic mesh-adaptation for moving boundaries.

Discretization in time is handled by the basic one-step Θ-scheme (3), which in general allows the selection of Crank-Nicolson ($\Theta = 0.5$) or the fully implicit Backward Euler-Scheme ($\Theta = 1$). The time discretization scheme in general can be represented by the following system:

For given $\vec{v}^{n-1}, p^{n-1}$ and $\Delta t = t_n - t_{n-1}$, lets approximate $\partial_t\vec{v}|_{t_n} \approx \frac{\vec{v}^n - \vec{v}^{n-1}}{\Delta t}$

$$\begin{cases} \varrho\Big(\vec{v}^n - \vec{v}^{n-1}\Big) + \Delta t\Theta\Big((\vec{v}^n\cdot\nabla)\vec{v}^n + \nabla\cdot\big(\mu(\nabla\vec{v}^n + (\nabla\vec{v}^n)^T)\big)\Big) + \Delta t\nabla p^n = \\ \qquad = \Delta t(\Theta - 1)\Big((\vec{v}^{n-1}\cdot\nabla)\vec{v}^{n-1} + \nabla\cdot\big(\mu(\nabla\vec{v}^{n-1} + (\nabla\vec{v}^{n-1})^T)\big)\Big) \\ \nabla\cdot\vec{v}^n = 0 \end{cases}$$ (Supplementary Eq. 3)

The system is then discretized in space using the Galerkin variational formulation of the Navier-Stokes equations. In our FEM framework we use the higher order Q_2/P_1 element pair for the spatial discretization, further aspects of the

FEM approach are described in more detail in our previous work [1]. Concerning the physical properties of the fluid, the density ρ is assumed to be constant, wheareas the viscosity μ is calculated at each cubature point according to the given shear-dependent rheological model. The system needs additional adjustment because of the presence of moving boundaries (the macro-scallop). These are treated by the so-called Fictitious Boundary Method (FBM) [1] which is a simple filter technique that decomposes the computational domain into a fluid and a solid subdomain in terms of the classification of the degrees of freedom. The fluid domain is then treated as if no FBM were applied, the solid subdomain interacts with the fluid subdomain by the imposed velocity Dirichlet boundary conditions (*i.e.* the rotational and translational velocity of the macro-scallop). The velocity of the macro-scallop is calculated by determining the hydrodynamic forces [1] that arise from the movement of the scallop. In order to improve the accuracy of the hydrodynamic force calculation we employ a grid-adaptation technique that is based on Laplacian smoothing using weights that force the vertices of the mesh to be concentrated at the fluid-solid interface (Supplementary Fig. 11).

Supplementary Note 2. Modulation of viscosity as underlying mechanism of propulsion

Let us consider an idealized tethered "scallop" (pump) composed of two infinite plates forming an angle α between them. The plates are co-rotating on a common axis with an arbitrary angular velocity ω. For simplicity we assume that the suspending medium is a Newtonian fluid. It is possible, however, to extend this solution and construct the asymptotic expansion corresponding to weakly non-Newtonian fluid (*e.g.* shear thinning or thickening) by the method of perturbations [2], however for our qualitative purposes the leading order solution suffices.

We consider the problem in the polar coordinates (r, φ). The solutions for the streamfunction $\psi(r, \varphi)$ in the Stokes approximation satisfy $\nabla^4 \psi = 0$. Following [3] and using an ansatz $\psi = r^2 f(\varphi)$ we find the solution for ψ the 'inner' (in between plates) and 'outer' regions, respectively:

$$\psi^{\text{in}}(r, \varphi) = -\frac{\omega r^2}{2} \frac{\sin 2\varphi - 2\varphi \cos 2\alpha}{\sin 2\alpha - 2\alpha \cos 2\alpha} = -\frac{\omega r^2}{2A_{\text{in}}} [\sin 2\varphi - 2\varphi \cos 2\alpha] \quad \text{(Supplementary Eq. 4)}$$

$$\psi^{\text{o}}(r, \varphi) = -\frac{\omega r^2}{2} \frac{\sin 2\varphi + 2(\pi - \varphi) \cos 2\alpha}{\sin 2\alpha + 2(\pi - \alpha) \cos 2\alpha} = -\frac{\omega r^2}{2A_{\text{o}}} [\sin 2\varphi - 2(\pi - \varphi) \cos 2\alpha] \quad \text{(Supplementary Eq. 5)}$$

where A_{in} and A_{o} are the functions of α. The velocity components $v_r = \frac{1}{r}\partial\psi/\partial\varphi$ and $v_\varphi = -\frac{1}{r}\partial\psi/\partial r$ can be readily obtained from the Supplementary Equations (4-5); for the inner region we have:

$$v_r^{\text{in}} = -\frac{\omega r}{A_{\text{in}}}[\cos 2\varphi - \cos 2\alpha], \quad v_\varphi^{\text{in}} = \frac{\omega r}{A_{\text{in}}}[\sin 2\varphi - 2\varphi\cos 2\alpha] \quad \text{(Supplementary Eq. 6)}$$

On the plates at $\varphi = \pm\alpha$, the boundary conditions are satisfied, *i.e.* $v_r^{\text{in}}(r, \pm\alpha) = 0$, $v_\varphi^{\text{in}}(r, \pm\alpha) = \pm\omega r$. The solution in the outer region has an analogous form. The streamlines (isolines of ψ) are depicted in Supplementary Fig. 13a-d for four different openings, $\alpha = 85.7°, 60°, 30°$ and $18°$. The corresponding vector velocity field is shown for illustration in Supplementary Fig. 13a. Note that the inner and outer solutions in Supplementary Equation (6) are singular at $\varphi \cong 129°$ and $51°$, respectively. This singularity is probably a consequence of nonphysical geometry as infinite plate result in two disconnected semi-infinite fluid domains. Considering finite plates should regularize the solution, however, the closed-form solution in such case is not readily available. Nevertheless, the infinite plate approximation should provide an accurate description of the flow far from the plates' ends at with for $\alpha < 50°$.

The components of the rate-of-strain tensor, D_{ij}, for the inner region can be found from Supplementary Equation (6) as

$$D_{rr}^{\text{in}} = \frac{\partial v_r^{\text{in}}}{\partial r} = -\omega A_{\text{in}}^{-1}[\cos 2\varphi - \cos 2\alpha]$$

$$D_{\varphi\varphi}^{\text{in}} = \frac{1}{r}\left(\frac{\partial v_\varphi^{\text{in}}}{\partial\varphi} + v_r^{\text{in}}\right) = \omega A_{\text{in}}^{-1}[\cos 2\varphi - \cos 2\alpha] = -D_{rr}^{\text{in}}$$

$$2D_{r\varphi}^{\text{in}} = \frac{1}{r}\left(\frac{\partial v_r^{\text{in}}}{\partial\varphi} - v_\varphi^{\text{in}}\right) + \frac{\partial v_\varphi^{\text{in}}}{\partial r} = 2\omega A_{\text{in}}^{-1}\sin 2\varphi$$

Next we calculate the (squared) *rate-of-strain* $\Gamma^2 = \frac{1}{2}D_{ij}D_{ij}$ (the shear rate $\dot{\gamma}$ is defined as $\dot{\gamma} = 2\Gamma$),

$$\Gamma^2 = \frac{1}{2}D_{rr}^2 + \frac{1}{2}D_{\varphi\varphi}^2 + D_{r\varphi}^2 = \omega^2 A_{\text{in}}^{-2}[1 - 2\cos 2\varphi\cos 2\alpha + \cos^2 2\alpha] \quad \text{(Supplementary Eq. 7)}$$

The result in Supplementary Equation (7) holds for the outer region with A_{in} being replaced with A_{o}. Note that for an infinite scallop Γ is not a function of r and depends solely on φ, *i.e.*, for an arbitrary opening Γ has a constant (but not equal) values along the plates inside and outside.

The corresponding plots of Γ/ω are given in Supplementary Fig. 14a-d for the same four openings α as in Supplementary Fig. 13. This figure illustrates the underlying physics of the phenomenon, *i.e.*, the plates play a role of the *concentrator* of the strain rate. Similarly to the electric capacitor concentrating the energy of the electric field in between two oppositely charged plates, the flapping plates concentrate the strain rate of the liquid sandwiched between the plates at small α. For Newtonian liquids this phenomenon is not important as both swimming and pumping are purely geometric, *i.e.* independent of time parameterization. Indeed, the fluid viscosity μ does not depend on the strain rate and remains constant so that over a full stroke such "scallop" would neither pump nor swim. For the non-Newtonian liquid, however, this phenomenon determines the essence of the effect. Since the apparent fluid viscosity μ_{app} is a function of the strain rate Γ, then the "scallop" modulates the viscosity of the suspending medium depending on contrast of the opening/closing frequencies. For shear thickening liquids the viscosity in the inner region could be considerably higher than that outside (compare Γ in Supplementary Fig. 14 c-d inside vs. outside). The dependence of the apparent viscosity on the strain rate distinguishes the liquid inside and outside the shells and results eventually in the free swimmer's locomotion or net momentum flux for a tethered pump.

To understand the origin of the motility heuristically, consider the fundamental solution for the low-*Re* flow driven by a point force of magnitude $F_{\text{p}}\delta(x)$ exerted on the fluid at the origin (this solution is known as *Stokeslet* [4]). One can argue that far from the object the origin of the force is not important – it equally can be submerged jet or flapping tethered "scallop". The properties of the source enters the solution integrally as a net momentum flux of magnitude F_{p}. If now the "scallop" becomes untethered, it will start moving with the characteristic speed, $V_{\text{s}} \sim F_{\text{p}}/\mu_0$. The crucial property of the swimmer is that it is propelled in the 'outer' liquid with low (approx. constant) viscosity μ_0, as in the outer region Γ is always small, see Supplementary Fig. 14 a-d, whereas the viscosity of the fluid sandwiched between the two plates $\bar{\mu}_{\text{app}}$ scales with the angular velocity of the *fast* (either closing or opening) stroke. The value of this internal viscosity determines the 'power' F_{p} of the source, $F_{\text{p}} \sim \bar{\mu}_{\text{app}}\omega$, whereas major contribution to $\bar{\mu}_{\text{app}}$ occurs at small opening α during the fast phase. Thus, the swimmer velocity V_{s} reduces to

$V_s/\omega l \sim \bar{\mu}_{app}/\mu_0$. This relation is in agreement with the estimate based on scaling arguments in the main text. The finite net displacement over a full stroke is due to modulation of the source strength, F_p, which depends on angular velocity ω in a nonlinear fashion via $\bar{\mu}_{app}(\omega)$.

Supplementary Note 3. Hysteresis of viscosity in shear thickening liquids

To understand the difference in the experimentally measured pre-factors β we re-write the power-law rheological model at low- and intermediate shear rates $\dot{\gamma}$ as

$$\mu_{app} \approx 0.7\left(\frac{\dot{\gamma}}{\dot{\gamma}_c}\right)^{-0.1}, \quad 0.1\ \mathrm{s}^{-1} \leq \dot{\gamma} \leq 1.5\ \mathrm{s}^{-1},$$

$$\mu_{app} \approx 0.8\left(\frac{\dot{\gamma}}{\dot{\gamma}_c}\right)^{2.34}, \quad 1.5\ \mathrm{s}^{-1} \leq \dot{\gamma} \leq 6.5\ \mathrm{s}^{-1},$$

where $\dot{\gamma}_c = 1.5\ \mathrm{s}^{-1}$ is the *critical shear rate at steady conditions* [5].

It has been found first in [5], then in [5, 6, 7] that the transition taking place at at $\dot{\gamma} = \dot{\gamma}_c$ possesses the properties of the phase transition of the first order. Namely, the transition is characterized by the *hysteresis of viscosity upon varying the shear rate*. In particular, in experiment with transient shear rate the value of viscosity depends on the prehistory of shear rate ramp: if the shear rate grows *from a low viscosity state*, the transition occurs at a higher critical shear rate than in steady state ($\dot{\gamma} > \dot{\gamma}_c$). Analogously, if the shear rate decreases *from a high viscosity state*, the transition takes place at a lower values of transient shear rate comparing to the steady state ($\dot{\gamma} < \dot{\gamma}_c$) [5].

The hysteresis of viscosity explains the asymmetry in propulsion upon exchanging between (fast) closing and opening strokes. Since opening and closing strokes are not identical (at opening the angle between plates changes from 10° up to 295° and at closing it decreases from 295° down to 10°). For (fast) opening, the shear rate decreases from a high viscosity state, and for the (fast) closing cycle the shear rate increases. In agreement with the hysteresis described in [5] the “scallop” propelled by (fast) opening stroke swims better than the one than relies on (fast) closing stroke.

The hysteresis of the shear thickening fluid was measured via a shear rate ramp loop test first from $1\ \mathrm{s}^{-1}$ up to $15\ \mathrm{s}^{-1}$ and then immediately from $15\ \mathrm{s}^{-1}$ down to $1\ \mathrm{s}^{-1}$. The loop was repeated for three times and the average is plotted in Supplementary Fig. 10. In agreement with the previsous works, the transition occurs at a higher critical shear rate when the shear rate increases from a low viscosity (blue squares), and vice versa a lower critical shear rate when decreasing (red triangles).

This hysteresis explains the reason that the average displacement per cycle of the backward stroke is larger than that of the forward stroke, under the same ratio of closing and opening (the absolute value of pre-factor $\beta = -0.014$ in Fig. 4c is larger than $\beta = 0.008$ in Fig. 4b). Specifically, the closing and opening strokes of the macro-scallop are not identical: at closing, as the gap between the shells changes from large to small, the shear rate increases and thus the transition of shear thickening occurs at a higher critical shear rate; vice versa at opening, the gap increases, the shear rate decreases, and the critical shear rate is lower. Therefore, in the opening half-cycle, the swimmer exhibits higher average viscosity than that in the closing half-cycle, and consequently results in better propulsion.

Supplementary References

1. Munster R, Mierka O, Turek S. Finite element-fictitious boundary methods (FEM-FBM) for 3D particulate flow. *Int J Numer Meth Fl* **69**, 294-313 (2012).
2. Normand T, Lauga E. Flapping motion and force generation in a viscoelastic fluid. *Phys Rev E* **78**, (2008).
3. Moffatt HK. Viscous and Resistive Eddies near a Sharp Corner. *Journal of Fluid Mechanics* **18**, 1-18 (1964).
4. Kim S, Karrila SJ. *Microhydrodynamics*. Butterworth-Heinemann (1991).
5. Laun HM, Bung R, Schmidt F. Rheology of Extremely Shear Thickening Polymer Dispersions (Passively Viscosity Switching Fluids). *J Rheol* **35**, 999-1034 (1991).
6. Nakanishi H, Nagahiro S, Mitarai N. Fluid dynamics of dilatant fluids. *Phys Rev E* **85**, 011401 (2012).
7. Deegan RD. Stress hysteresis as the cause of persistent holes in particulate suspensions. *Phys Rev E* **81**, 036319 (2010).
8. Keim NC, Garcia M, Arratia PE. Fluid elasticity can enable propulsion at low Reynolds number. *Phys Fluids* **24**, 081703 (2012).
